# Dramatic decreases of malaria transmission intensities in Ifakara, south-eastern Tanzania since early 2000s

**DOI:** 10.1186/s12936-018-2511-2

**Published:** 2018-10-16

**Authors:** Marceline F. Finda, Alex J. Limwagu, Halfan S. Ngowo, Nancy S. Matowo, Johnson K. Swai, Emmanuel Kaindoa, Fredros O. Okumu

**Affiliations:** 1 Environmental Health and Ecological Science Department, Ifakara Health Institute, P. O. Box 53, Ifakara, Tanzania; 2 School of Public Health, Faculty of Health Sciences, University of the Witwatersrand, Johannesburg, South Africa; 3 University of Basel, Basel, Switzerland; 4 Swiss Tropical and Public Health Institute, Basel, Switzerland; 5 Institute of Biodiversity, Animal Health and Comparative Medicine, University of Glasgow, Glasgow, G12 8QQ UK

## Abstract

**Background:**

Ongoing epidemiological transitions across Africa are particularly evident in fast-growing towns, such as Ifakara in the Kilombero valley, south-eastern Tanzania. This town and its environs (population ~ 70,000) historically experienced moderate to high malaria transmission, mediated mostly by *Anopheles gambiae* and *Anopheles funestus*. In early 2000s, malaria transmission [*Plasmodium falciparum* entomological inoculation rate (P*f*EIR)] was estimated at ~ 30 infectious bites/person/year (ib/p/yr). This study assessed the P*f*EIR after 15 years, during which there had been rapid urbanization and expanded use of insecticide-treated nets (ITNs).

**Methods:**

Randomly-selected 110 households were sampled across Ifakara town and four adjacent wards. Mosquitoes were trapped nightly or monthly (June.2015–May.2016) using CDC-light-traps indoors, Suna^®^ traps outdoors and human landing catches (HLC) indoors and outdoors. All *Anopheles* mosquitoes were morphologically identified and analysed by ELISA for *Plasmodium* circumsporozoite proteins. Mosquito blood meals were identified using ELISA, and sub-samples of *An. gambiae* and *An. funestus* examined by PCR to distinguish morphologically-similar siblings. Insecticide resistance was assessed using WHO-susceptibility assays, and some *Anopheles* were dissected to examine ovariole tracheoles for parity.

**Results:**

After 3572 trap-nights, one *Plasmodium*-infected *Anopheles* was found (an *An. funestus* caught outdoors in Katindiuka-ward by HLC), resulting in overall P*f*EIR of 0.102 ib/p/yr. Nearly 80% of malaria vectors were from Katindiuka and Mlabani wards. *Anopheles gambiae* densities were higher outdoors (64%) than indoors (36%), but no such difference was observed for *An. funestus*. All *An. funestus* and 75% of *An. gambiae* dissected were parous. *Anopheles gambiae* complex consisted entirely of *Anopheles arabiensis*, while *An*. *funestus* included 84.2% *An*. *funestus* s.s., 4.5% *Anopheles rivulorum*, 1.4% *Anopheles leesoni* and 9.9% with unamplified-DNA. *Anopheles gambiae* were susceptible to bendiocarb and malathion, but resistant to pyrethroids, DDT and pirimiphos-methyl. Most houses had brick walls and/or iron roofs (> 90%), and 52% had screened windows.

**Conclusion:**

Malaria transmission in Ifakara has decreased by > 99% since early-2000s, reaching levels nearly undetectable with current entomological methods. These declines are likely associated with ITNs use, urbanization and improved housing. Remaining risk is now mostly in peri-urban wards, but concerted efforts could further decrease local transmission. Parasitological surveys are required to assess actual prevalence, incidence and importation rates.

## Background

Globally, human populations and settlements are undergoing rapid expansion, concurrent with speedy urbanization [1]. In the 1950s, less than one-third of global population lived in urban areas, but this had increased to 54% by 2014 and will likely exceed two-thirds by 2050 [1]. Most of these changes are expected to occur in developing countries in Africa and Asia [1, 2]. In Tanzania, about 33% of people currently live in urban areas, compared to just 20% in 2000 [3, 4].

Malaria burden has significantly declined worldwide over the past two decades, largely because of deliberate control efforts like scale-up of insecticide-treated nets (ITNs) and improved treatments [5] as well as due to socio-economic developments [6, 7]. Urbanization is one of the major aspects of development that significantly impacts malaria transmission [7–9]. Processes of urbanization reduce malaria transmission primarily because urban environments lack suitable *Anopheles* breeding habitats [8–10]. Additionally, urban settings generally have better access of health services and improved housing as well as greater education and awareness than rural areas [7, 9]. In a 2013 analysis, Tatem et al. concluded that ongoing urbanization in endemic regions would cause further declines in malaria transmission, and that these declines would be synergized by the deliberate scale-up of core malaria control tools [7].

Ifakara town, in the Kilombero river valley in the mostly agricultural Morogoro region, is among the fast growing towns in Tanzania. The town is surrounded by rain forests, wetlands and vast savannah lands, making it an attractive setting for both farmers and pastoralists. It is also a key trading hub in the region, mostly for farm and animal products. Towns such as Ifakara that appear in the middle of low-lying tropical floodplains present unique scenarios for assessing spatial and temporal variations of malaria transmission intensities [i.e. entomological inoculation rates (EIR), measured as number of infectious bites/person/year (ib/p/yr)]. Historical measures of EIR in Ifakara date back to 1965, when the town still had just one-fifth of its current population, and when Freyvogel and Kihaule conducted a small survey of *Anopheles* mosquitoes in the area [11]. Though parasite assessment techniques at the time were still fairly insensitive compared to options available today, Freyvogel and Kihaule reported monthly “apparent inoculation rates” for both *Anopheles gambiae* and *Anopheles funestus*, the two predominant malaria vectors at the time [11]. The “apparent inoculation rate” represented how frequently a single person was likely to get infected, and was estimated based on factors such as proportion of mosquitoes with sporozoites in their salivary glands upon dissection, gonotrophic cycles of the mosquitoes, and estimated daily biting rates (computed as ratio of total indoor mosquito catches and number of sleepers in collection room). In later years, there were multiple EIR assessments in the surrounding villages, including which reported 329 ib/p/yr from early 1990s in Namawala, a rural community 30 km away from the Ifakara town [12]. Because of the differences in assessment methods, it is difficult to directly compare these historical estimates against measures obtained after 1990s onwards.

In 2003, Drakeley et al. published findings of another entomological survey that assessed malaria transmission intensities in Ifakara, which at that time was still a peri-urban area surrounded by very high transmission villages in the Kilombero valley [13]. In the years preceding this report, the team had conducted fortnightly mosquito collections using CDC-light traps inside 32 randomly selected houses in Ifakara area. They used two different methods to estimate the P*f*EIR. First was the standard method, which involved calculations of the product of sporozoites rate (SR) and human-biting rate (BR), and the second method involved calculation of the ratio of sporozoites-positive mosquitoes to the number of nightly catches. Drakeley et al. estimated an EIR of 31 ib/p/yr using the standard method and 29 ib/p/yr using the alternative method [13].

This study aimed to assess malaria transmission intensities in Ifakara town and its adjacent wards, more than a decade after the assessment by Drakeley and colleagues. This new study employs similar mosquito sampling techniques, but with addition of indoor/outdoor comparisons of transmission risk, and an inclusion of an odour-bated trap to sample outdoor host-seeking vectors. Similar methods were also used for calculating and reporting P*f*EIR as used by Drakeley et al. [13]. The primary objective was to assess the extent to which malaria transmission in the study area had changed over the past 15 years, considering the rapid growth in population [2, 14], urbanization trends [15] and the sustained use of effective malaria treatment as well as prevention measures such as ITNs [16–19].

## Methods

### Study site

**Fig. 1:**
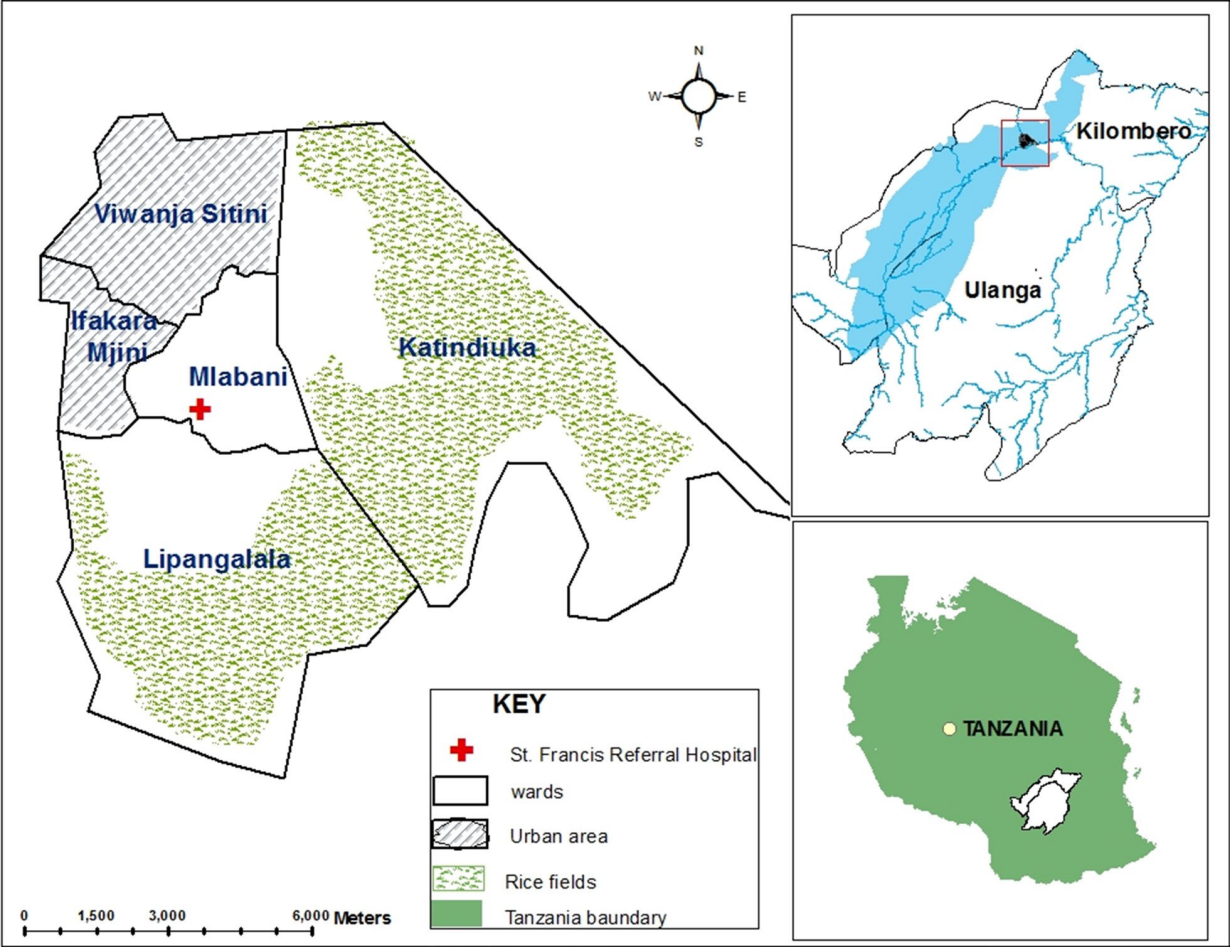
Map of the study areas showing five wards that make up the Ifakara town

This study was conducted in Ifakara town and its adjacent wards located in the Kilombero river valley, in south-eastern Tanzania (Fig. 1). The small but rapidly-growing town lies at 8.1336° South and 36.855° East. It has an average altitude of 270 m above sea level, annual rainfall of 1200–1800 mm, annual temperature range between 20 and 32 °C, and relative humidity between 51 and 71%. The Ifakara town area is administratively divided into five wards, namely Ifakara Mjini (Ifakara town centre), Katindiuka, Lipangalala, Mlabani and Viwanja Sitini. In 2012, when the last national census was conducted, Ifakara town had a population of 55,956 people [20]. Given the estimated country population growth rate of 3.1%, the town currently has an estimated population of about 67,345 people as of 2018 [2, 3]. In 2002 when Drakeley and colleagues had conducted the initial survey, Ifakara town had a population of 45,518 people [20], and between then and now the population has increased by nearly 50%. Ifakara Mjini and Viwanja Sitini, located on the western part of the town, are the most urban of the five wards, and the inhabitants are largely small business owners. These two wards make up of about 56% of the total population. On the other hand, Katindiuka and Mlabani, located on the eastern part of the town, are the most rural and least densely populated of the five wards and are surrounded by seasonal rice farms. This study was conducted in all the five wards (Fig. 1) for a period of 12 months between June 2015 and May 2016.

Recent studies conducted in neighboring villages in the valley have shown that primary malaria vector species include *Anopheles* *arabiensis* and *An.* *funestus*, with the latter mediating > 80% of the ongoing transmission [21]. Other *Anopheles* mosquitoes found in the area are *Anopheles coustani, Anopheles pharoensis, Anopheles squamosus, Anopheles ziemanni, Anopheles maculipalpis* and *Anopheles wellcomei*. Malaria vector densities are highest during the rainy season, with peaks between March and May, though *An. funestus* densities may peak after the rains [22]. The major vector control intervention is long-lasting insecticide-treated nets (LLINs), mostly provided freely by the Government; the last such mass-distribution of the nets having been in 2016.

### Sampling procedures

Geo-referenced house-hold database obtained from Ifakara Health and Demographic Surveillance Systems (IHDSS) [23] covering the five wards was used to select the households for this study. A total of 22 households were randomly selected from each of the five wards, ensuring approximately equal representation from each *Balozi* (an administrative cluster consisting of 10–100 households). In each ward, two households were visited for 5 days every month for 12 months, and mosquito sampling was done both indoors and outdoors. The other 20 households were each visited twice every month, once for indoors and once for outdoors mosquito sampling. Altogether a total of 3600 trap-nights were planned in 110 households in the five wards for a period of 12 months between June 2015 and May 2016. The full sampling plan is shown in Table 1.

**Table 1:** Illustration of complete sampling plan for the vector surveillance and estimation of malaria transmission in Ifakara town and its environs

Trap type	Position	Sample station	Frequency	Number of houses	Number of trap nights
Human landing catches	Indoors	Sentinel	Hourly collections, 5 nights per month	10	600
Human landing catches	Outdoors	Sentinel	Hourly collections, 5 nights per month	10	600
CDC light traps	Indoors	Random	Nightly collections, 1 night per month	100	1200
Suna^®^ Traps	Outdoors	Random	Nightly collections, 1 night per month	100	1200

The table shows the different trapping methods used, their frequency of use, position of use (indoors/outdoors), and whether the methods were used in sentinel or randomly selected households. The sampling plan does not include the additional collections done to assess parity

### Mosquito collections indoors and outdoors

**Fig. 2:**
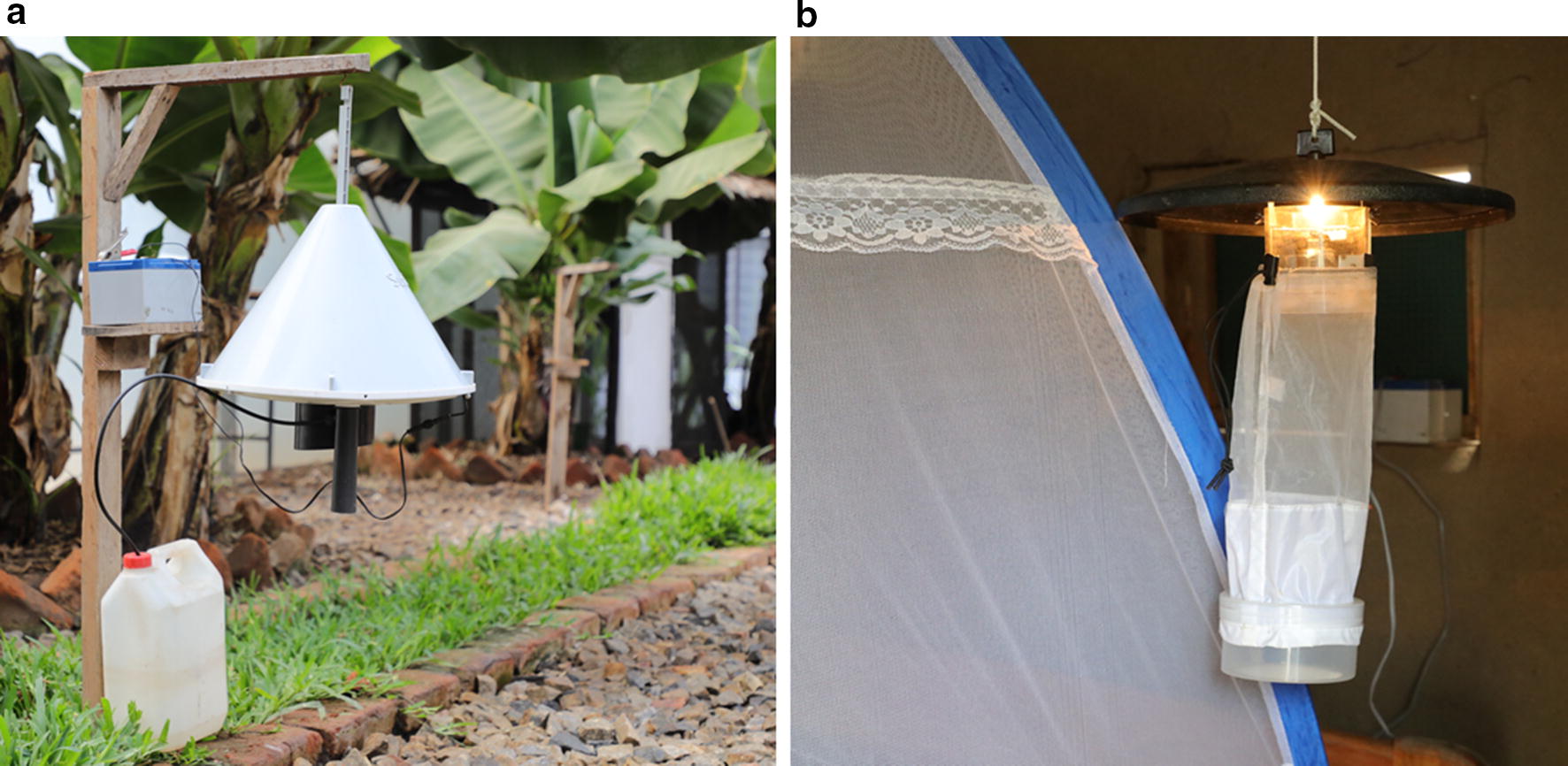
Pictures of sampling methods used: **a** SUNA trap placed outdoors and **b** CDC-light trap placed near occupied bed net

Indoor mosquito catches were done using standard Centres for Disease Control (CDC) light traps [24], set near human-occupied bed nets (Fig. 2). The light traps were suspended at the foot-end of the bed, approximately 1.5 m above ground [25]. The traps were set between 18:00 h and 06:00 h each sampling night in a room with at least one person sleeping under an intact bed net. Where no intact nets were available in the sampling room, one was provided freely to each consenting household. The traps were retrieved each morning starting 06:00 h; the field team taking approximately 30 min to collect all traps from all household stations. Outdoor mosquito collections were done using odour-baited Suna^®^ Traps [26] (Fig. 2). The Suna^®^ traps were suspended 30 cm above the ground, and 5–10 m from the selected house. The traps were baited using a combination of Ifakara synthetic lure [27] dispensed in pellets and CO_2_ gas produced from yeast-molasses fermentation [28]. Like the CDC light traps, the Suna^®^ traps were set between 18:00 and 06:00 h and retrieved at 06:00 h each subsequent morning. To minimize any direct competition between the indoor and outdoor traps, the sampling plan was such that the Suna^®^ and CDC light traps where never at the same house at any night, instead being placed in different nights. This specific dataset was, therefore, also not used for comparing indoor vs. outdoor biting rates. These CDC light trap and Suna™ trap collections were done consistently in the 20 selected houses in each ward, totaling 100 households, each sampled monthly for indoors collections and monthly for outdoor collections.

To assess and compare indoor and outdoor biting preferences and nightly biting patterns, additional collections were done in 10 households using human landing catches method, working with consenting and trained adult male volunteers. In each of the 10 households, two adult male volunteers sat either indoors or outdoors and collected mosquitoes hourly, working for 45 min and resting for 15 min every hour. The collections were done between 18:00 and 06:00 h and each household was sampled for five nights every month for 12 months. In the 5-day period of HLC, the collections were done every other day, to allow volunteers a day of resting between the collections. All collected mosquitoes were kept in paper-cups and labeled to show date, hour of collection and household identification codes.

At the end of this survey, additional adult malaria vectors [*An. gambiae* sensu lato (s.l.) and *An. funestus*] were collected using CDC light traps, and used to assess parity rates and estimate physiological age of the mosquito populations in the area [29]. These additional collections were done for 1 month in 10 households per ward in three of the five study wards, i.e. Katindiuka, Mlabani, and Lipangalala.

### Sorting and laboratory analysis of mosquito samples

Each morning, mosquitoes collected the previous night were killed using 70% ethanol and sorted by sex and taxa using morphological identification keys, primarily to separate *Anopheles* mosquitoes from other genera, and to identify major malaria vector groups [30, 31]. Abdominal status of the female *Anopheles* mosquitoes was assessed and recorded as unfed, blood-fed or gravid. *Anopheles* mosquito samples were packed in labeled plastic micro-centrifuge tubes (Eppendorf^®^) with silica gel as desiccant and sent to the Ifakara Health Institute laboratory for further analyses, including: (a) species identification using Polymerase Chain Reaction (PCR) [32, 33], (b) detection of *Plasmodium* parasites in primary malaria vectors using Enzyme Linked Immunosorbent Assays (ELISA) [34], and (c) identification of mosquito blood meals using ELISA [35]. The specific methods used for the laboratory analyses were as follows:

#### PCR assays for species identification

DNA was first extracted from the mosquito hind legs. For *An. gambiae* s.l., PCR amplification was done for the species-specific nucleotide sequences of the ribosomal DNA (rDNA) intergenic spacer regions (IGS) in a 25 µl reaction volume of PCR mixture following methods first described by Scott et al. [33]. For *An. funestus* group mosquitoes, methods developed by Koekemoer et al. [32] were used to distinguish between up to five members of the *An. funestus* group, including the three that have been previously described in villages around the current study area, i.e. *An. rivulorum, An. funestus* sensu stricto (s.s.) and *An. leesoni* [21]. Here, amplification was done for the species-specific non-coding regions of the internally transcribed spacer 2 (ITS2) region on the rDNA. After amplification, the post-PCR amplicons for both the *An. gambiae* and *An. funestus* were analysed by electrophoresis in agarose gel stained with ethidium bromide. Visible DNA bands were photographed under ultraviolet light using Kodak Gel Logic 100 imaging system.

#### ELISA assays for parasite detection in mosquito salivary glands

To detect *P. falciparum* circumsporozoite proteins (CSP) in the salivary glands of the mosquitoes, ELISA assays were done on pooled samples separately for *An. gambiae* s.l. and *An. funestus.* Sample pooling was done by household ID, hour and date of collection and sampling method, and did not exceed 10 individual mosquitoes. Where fewer than 10 mosquitoes of one species complex were collected by a given method on a given hour or date at a given house, then the pool size was also less than 10. The optical density of post-ELISA lysate was measured at 405–414 nm after 45 min using ELISA plate readers. ELISA lysates for all postive samples were boiled at 100 °C for 15 min, to eliminate any false positives, usually associated with heat-labile protozoans in *Anopheles* mosquitoes, especially those with previous bovine blood-meals [36–38].

#### ELISA assays for identification of mosquito blood meal sources

Abdomens of all the blood-fed mosquitoes were screened to detect antigens for human, bovine, goat, dog and chicken blood antigens, as these are the most likely blood-meal sources in the area. Anti-IgG antibodies from each host were used to detect host antigen in the blood meal of the mosquito based on the method described by Beier et al. [35].

### Assessment of insecticide susceptibility rates in malaria vectors

The World Health Organization (WHO) standard insecticide susceptibility assays [39] were performed on female adult mosquitoes raised from larvae sampled from two of the five study wards, i.e. Katindiuka and Viwanja Sitini. These assays were possible only with *An. arabiensis,* but not *An. funestus* because of the low densities and cryptic nature of *An. funestus* aquatic habitats in the area. Mosquito larvae were reared to adult stage under standard insectary conditions (temperature of 27 ± 2 °C and relative humidity of 70–90%) inside the Ifakara Health Institute mosquito laboratory, the VectorSphere. The larvae could feed on the mud and some algae brought together during collection but small quantities of Tetramin^®^ baby fish (Tetra, Melle, Germany) food was also added. Each morning pupae were collected and placed in plastic cups inside netting cages measuring 30 m × 30 m × 30 m. Upon emerging, adult mosquitoes were fed on 10% glucose solution prior to exposure to insecticides. Non-blood fed females of *An. arabiensis* (2–5 days old) were used in the assays, following the WHO test procedures [39]. The bioassays were done on 0.05% lambda cyhalothrin, 0.05% deltamethrin, 0.75% permethrin, 4% DDT, 0.1% bendiocarb, 0.25% pirimiphos-methyl and 5% malathion. Ifakara strain of *An. gambiae* s.s. was used as a reference susceptible strain. Four replicates of the exposure were done per insecticide, and each exposure used 25 mosquitoes. Mortality was recorded 24 h post-exposure.

### Assessment of parity status

Additional adult malaria vectors were collected from inside the houses using miniature CDC light traps [24] to determine their physiological age [29]. These mosquitoes were collected from 10 households in three of the five wards: Katindiuka, Mlabani, and Lipangalala. Each morning, the mosquitoes were killed by refrigeration and immediately dissected under a stereo light microscope. The dissected ovaries were observed on 10× magnification under a compound microscope, to determine parity status by recording presence of stretched ovariole tracheoles (parous females) or coiled tracheolar skeins (nulliparous females), as detailed in the WHO Practical Manual for Entomology [29].

### Household characterization

Additional information on the type of construction of each of the 110 houses was collected. This included type of construction material used for the roof and walls, materials used to cover the windows and doors, types of animals the houses kept and whether the houses had access to electricity.

### Data processing, analyses and estimation of transmission intensities

Entomological data was recorded in standardized data forms used at Ifakara Health Institute. The data was quality-checked and double-entered into a Microsoft Excel database by two data entry clerks. The two data sets were compared to correct any errors, after which a single clean dataset was produced for further analysis.

#### Generalized linear mixed models for mosquito catch data

Data analysis was done using R statistical software version 3.3.2 [40]. Generalized linear mixed models (GLMM) [41] were used in analysis of mosquito densities by different study wards, mosquito trapping methods and position, i.e. indoors versus outdoors in cases where HLC had been used. In all analysis, study wards were treated as fixed factor while experimental day was treated as random term, controlling for seasonal variations. Initially, all the models were fitted using Poisson distribution models for count data with log-link functions, but due to poor-fitting and over dispersion, negative binomial distributions were fitted using *glmmadmb* (generalized linear mixed model automatic differentiation model builder) package [42], which allowed assessment of the zero inflations in the count data. Model testing was done using *Akaike Information Criterion* (AIC), by sequentially selecting models with lower AIC values. Graphs were produced using a *ggplot2* package in R [43]. The mosquitoes caught using HLC were also analysed for nightly biting patterns and graphically presented to illustrate time of night when peak biting occurred.

#### Calculating PfEIR

Similar approaches as used by Drakeley et al. [13] were applied to calculate *Plasmodium* infection rates (sporozoites rates) and P*f*EIR. These analyses were initially done for each administrative ward, each month and each trap type, but was later pooled so that there was a single estimate of infection rate in all mosquitoes collected. Human-biting rates (HBR) were measured directly from human landing collections made indoors. The *P. falciparum* sporozoites rate (P*f*SR) was calculated by dividing the number of *Plasmodium*-positive mosquitoes by the total number of mosquitoes examined by ELISA, and this was expressed as a percentage. *Plasmodium falciparum* entomological inoculation rate (P*f*EIR) was then calculated considering the two methods previously used by Drakeley et al. [13] as follows:

The first was the standard method of calculating P*f*EIR as a product of sporozoites rates and human-biting rates; i.e., C*(Number of sporozoites positive mosquitoes/Number of mosquitoes tested) × (Number of mosquitoes collected/Number of trap nights) × 365 [44], where, C is the relative efficiency coefficient for the relationship between CDC-light trap catches and human landing catches. The second method involved calculation of the ratio of sporozoites positive mosquitoes and number of nightly catches; i.e., C*(no of sporozoites positive mosquitoes/number of trap nights) × 365 [13]. In this study all collected *An. gambiae* and *An. funestus* mosquitoes collected were analysed, therefore minimal variation in the two P*f*EIR estimates were expected. Although multiple trap types were used, P*f*EIR was only detectable using HLC, since the only sporozoite positive mosquito was collected using this method.

In the analyses by Drakeley et al., they had assumed that traps with extraordinarily large numbers of catches compared to the average, would be consisting mostly of young nulliparous mosquitoes, which are naturally sporozoites negative [45]. Such trap collections were therefore all considered “tested” but with negative CSP result. In this study however, mosquito samples were pooled in maximum pool size of 10 mosquitoes per assay, and all collected samples were analysed so that any biases associated with the said assumptions were effectively eliminated. In the Drakeley et al. analysis [13], a value of 1.605 (equivalent to 0.62 relative efficacy of CDC-light traps to HLC) was used based on previous studies by Charlwood et al. [45]. However, in recent estimates by Kaindoa et al. [21], values of 0.3 for *An. funestus* and 0.68 for *An. gambiae* s.l. were used based on 2004 estimates by Okumu et al. [46] for the relative efficiencies of CDC-light trap catches relative to HLC. However, in a wide-ranging review of the estimates of CDC light trap and HLC catches across Africa, Briët et al. [47] concluded that there is no need to do these conversions, and that the CDC-Light trap estimate may be used as is. In this study therefore, P*f*EIR estimates were calculated without adjustments. The coefficient, C, in the P*f*EIR equations was fixed at 1, assuming all the trap types had similar efficiency across space and time. Nonetheless, any adjustments in human biting rates would be insignificant given no sporozoites positive mosquitoes were collected by any method other than HLC.

#### Assessing mosquito host preferences

Contributions of different blood meal sources in the malaria vectors were estimated as percentages of total mosquitoes assayed.

#### Analysis of spatial patterns in vector abundance

Mosquito densities and distribution across the study area was assessed for evidence of spatial associations and clustering. Spatial patterns of total nightly mosquito densities per house were examined using ArcGIS 10.4 spatial analyst tool (ESRI, USA). The Getis-Ord Gi* statistic [48, 49] in Arc-GIS was used to identify households with significant clustering of high densities of the two main malaria vectors; *An. arabiensis* and *An. funestus*. Clusters depicting both the high vector density foci and low-vector density foci were identified and their statistical significance determined at Gi* P value ≤ 0.05, and Gi* Z score ≥ 1.96. Spatial relationships between households were assumed to be inversely related, such that houses far apart were more likely to differ in vector densities than households near one another. The analysis was done assuming Euclidean distances between neighboring mosquito sampling stations [48, 49].

## Results

### Overall mosquito densities

A total of 264,012 mosquitoes were collected in the 3572 trap nights, considering all trap types. The collections comprised of 257,399 culicines (97.5%) and 8613 *Anopheles* mosquitoes (3.3%). The *Anopheles* included 7795 *An. arabiensis* (90.5%), 400 *An. funestus* (4.6%), 310 *An. coustani* (3.6%) and 112 of other *Anopheles* species (1.3%).

**Fig. 3:**
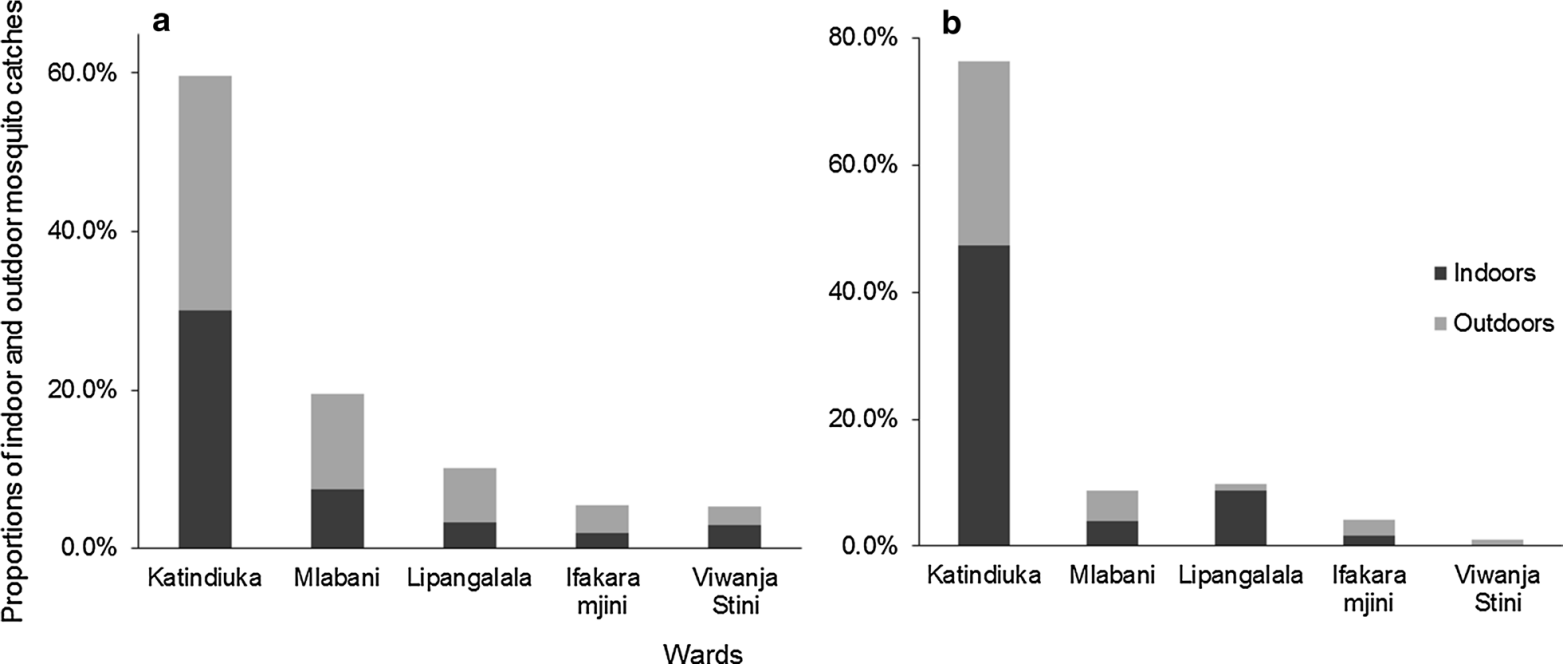
Proportions of indoor and outdoor mosquito catches in the sampling stations for; **a** *An. arabiensis*; **b** *An. funestus.* This figure illustrates only data collected using human landing catches

**Fig. 4:**
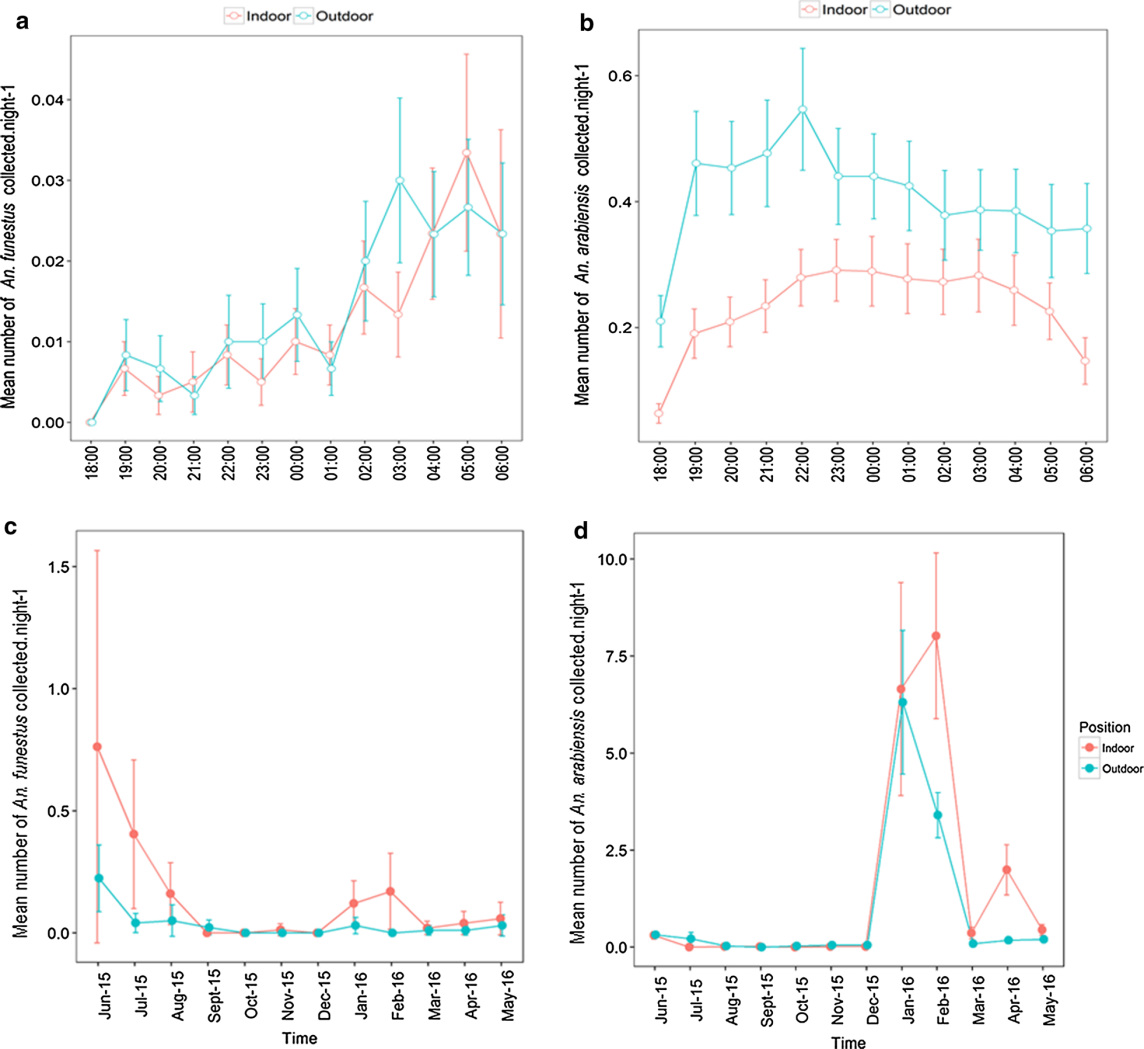
Distribution of mosquitoes in Ifakara town: **a** hourly distribution of *An. funestus*; **b** hourly distribution of *An. arabiensis*; **c** monthly distribution of *An. funestus* and **d** monthly distribution of *An. arabiensis*

Indoor and outdoor densities of the mosquitoes were compared using the HLC method. Overall, *An. gambiae* densities were higher outdoors (64%) than indoors (36%), while only marginal differences were observed in *An. funestus*, 46% of which were indoors and 54% outdoors (Table 2). Similar patterns were observed across the five wards (Fig. 3). The nightly biting patterns of mosquitoes collected by HLC in the ten sentinel houses in each ward are also shown in Fig. 4, which also shows that indoor collections were significantly lower than outdoor collections for *An. arabiensis* but not *An. funestus* throughout the night.

**Table 2:** Number of mosquitoes of different species collected indoors and outdoors by the different trapping methods in both sentinel and random stations across the study area

Trapping method	No. trap nights	No. houses	Mosquito species	No. mosquitoes indoors (%)	No. mosquitoes outdoors (%)	Total
Human landing catches	1200	10	*Anopheles arabiensis*	1807 (36.2%)	3187 (63.8%)	4994
			*Anopheles funestus*	94 (46.3%)	109 (53.7%)	203
			Other *Anopheles* species	32 (9.5%)	305 (90.5%)	337
			*Mansonia* spp.	461 (30.6%)	1048 (69.4%)	1509
			Culex spp.	68,582 (54.3%)	57,716 (45.7%)	126,298
CDC-light traps	1192	100	*Anopheles arabiensis*	1736 (100%)	NA	1736
			*Anopheles funestus*	158 (100%)	NA	158
			Other *Anopheles* species	27 (100%)	NA	27
			*Mansonia* spp.	832 (100%)	NA	832
			Culex *spp.*	51,958 (100%)	NA	51,958
Suna^®^ traps	1180	100	*Anopheles arabiensis*	NA	1065 (100%)	1065
			*Anopheles funestus*	NA	39 (100%)	39
			Other *Anopheles* species	NA	54 (100%)	54
			*Mansonia* spp.	NA	1551 (100%)	1551
			Culex spp.	NA	73,251 (100%)	73,251

### Spatial and temporal variations of malaria vector biting risk

**Fig. 5:**
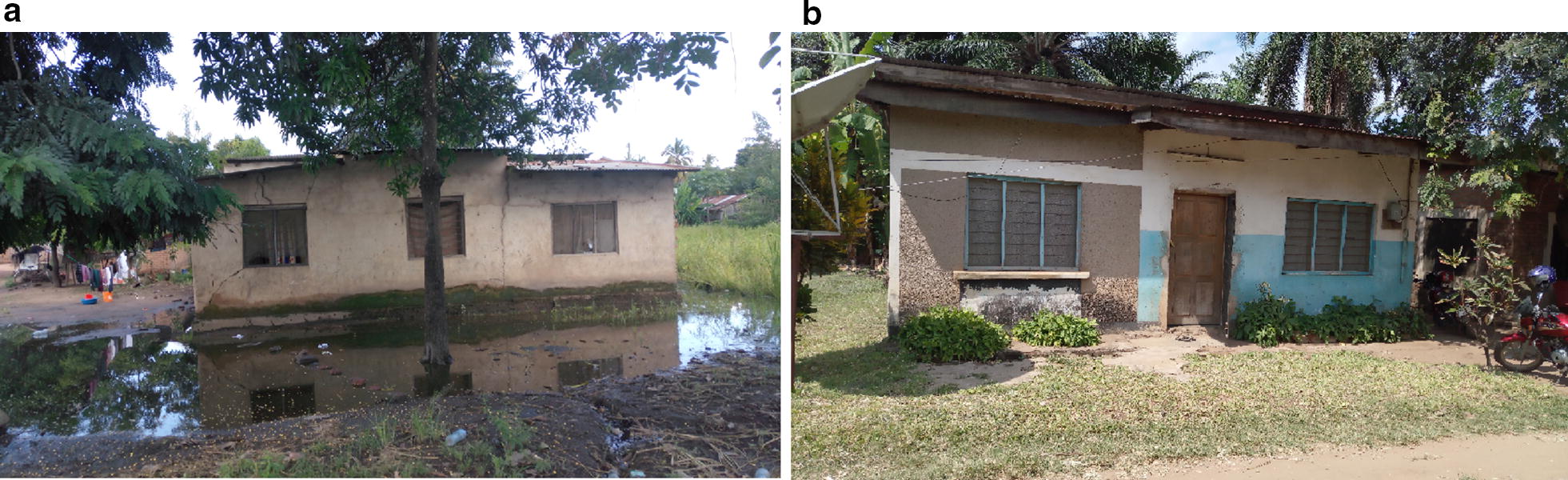
Examples of house structures and locations in Ifakara town: **a** a typical house in Katindiuka ward, showing aquatic breeding habitats of *Anopheles* mosquitoes (rice fields and water ponds) nearby and **b** a typical house in the more urban settings of Ifakara town

**Fig. 6:**
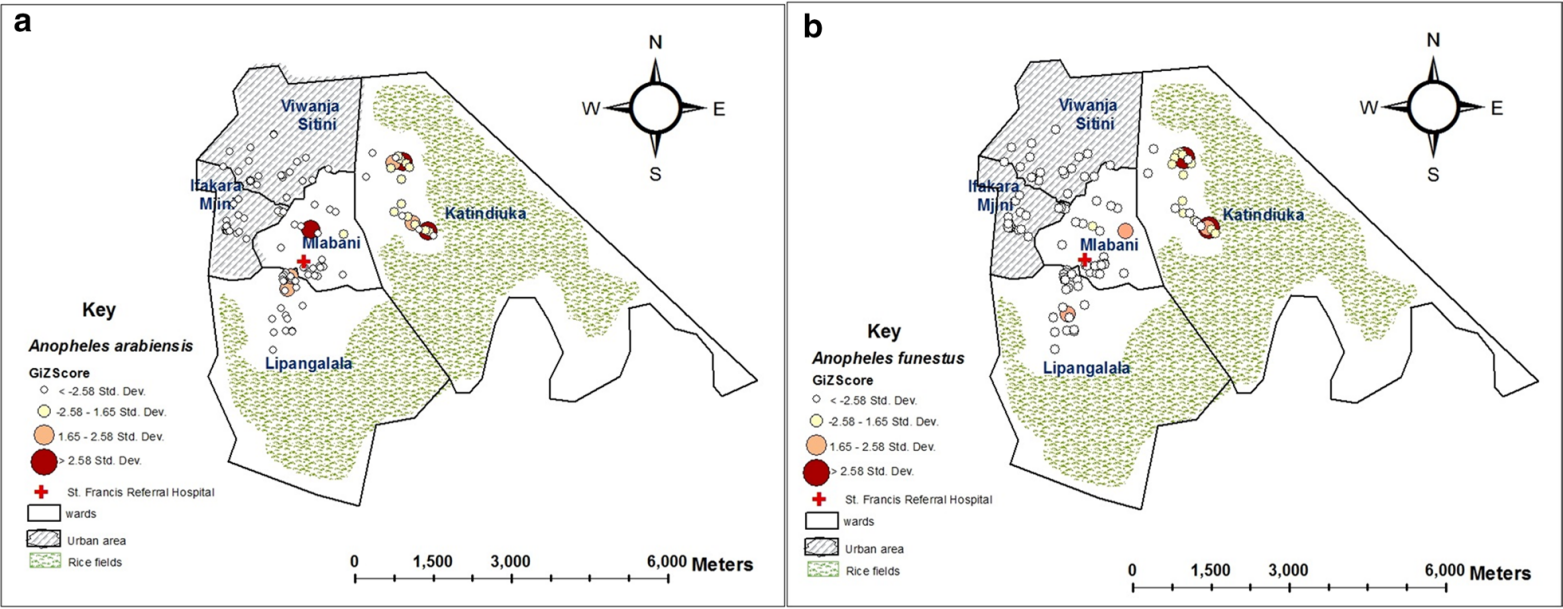
Map showing distribution of **a** *An. arabiensis* and **b** *An. funestus* within the five wards of Ifakara town. All clusters depicting areas with households where the highest densities are most spatially concentrated were first identified, after which statistically significant ones were determined at level of Gi* P value ≤ 0.05, and Gi* Z score ≥ 1.96. The actual Getis-Ord Gi* statistics are provided to illustrate areas with maximum and minimum vector densities

Over the entire sampling period, 78.5% of all the primary malaria vectors were collected from two of the five wards, i.e. Katindiuka and Mlabani, which were the peri-urban parts. The remaining 21.5% were collected from the rest of the study area, i.e. Ifakara Mjini, Lipangalala and Viwanja Sitini, which were the more urbanized parts. In particular, Katindiuka ward, with houses constructed very close to mosquito breeding habitats (Fig. 5) had the higher densities of both *An. arabiensis* and *An. funestus* compared to the other wards (Table 3). Within Katindiuka ward itself, highest densities were in households closer to the adjacent rice fields on the eastern side of the ward, compared to households at the centre and on the western side (Fig. 6). This distribution pattern was observed for both *An. arabiensis* and *An. funestus,* although *An. funestus* were caught at a much lower densities (Table 3). Densities of *An. arabiensis* were highest between January and May, peaking in February, while the *An. funestus* densities were highest between May and August, peaking in June (Fig. 4).

**Table 3:** Mean numbers of mosquitoes of different species caught each night in the four adjacent wards of Ifakara, relative to the numbers caught in Ifakara Mjini (the main town centre)

Species	Wards	Mosquito catches indoors				Mosquito catches outdoors			
		Total no. mosquitoes	Adjusted means [UC–LC]	RR [UC–LC]	P-values	Total no. mosquitoes	Adjusted means [UC–LC]	RR [UC–LC]	P-values
*Anopheles arabiensis*	Ifakara Mjini	154	0.42 [0.25–0.70]	1	N/A	269	0.29 [0.17–0.51]	1	N/A
	Katindiuka	2337	5.81 [3.53–9.56]	13.8 [6.9–27.6]	< 0.001	2318	3.28 [1.98–5.42]	11.2 [5.33–23.7]	< 0.001
	Lipangalala	254	0.16 [0.09–0.30]	0.4 [0.2–0.8]	< 0.05	531	0.10 [0.05–0.19]	0.3 [0.14–0.79]	< 0.05
	Mlabani	580	0.22 [0.12–0.39]	0.5 [0.2–1.1]	0.084	939	0.31 [0.18–0.53]	1.1 [0.48–2.29]	0.898
	Viwanja Sitini	225	0.64 [0.38–1.07]	1.5 [0.7–3.1]	0.257	188	0.54 [0.32–0.92]	1.9 [0.87–4.00]	0.11
*Anopheles funestus*	Ifakara Mjini	7	0.002 [0–0.02]	1	N/A	10	0.02 [0.01–0.05]	1	N/A
	Katindiuka	189	0.49 [0.25–0.97]	212.7^a^ [24.1–1881]	< 0.001	116	0.10 [0.05–0.18]	4.5 [1.68–14.78]	< 0.001
	Lipangalala	35	0.03 [0.01–0.10]	14.5^a^ [1.6–132.0]	< 0.05	4	0 [0–0]	0 [0–0]	0.802
	Mlabani	16	0.01 [0.003–0.04]	5.0 [0.5–53.9]	0.184	19	0.03 [0.01–0.07]	1.4 [0.39–4.83]	0.627
	Viwanja Sitini	1	0.002 [0–0.02]	1.1 [0.1–21.2]	0.942	3	0.01 [0.002–0.03]	0.4 [0.07–2.24]	0.298

The catch densities are also illustrated in maps in Fig. 6

^a^Excessively dispersed data not tractable by the generalized linear mixed models, mostly because of very low-densities of *An. funestus* mosquitoes

### Sibling species of primary malaria vectors

A total of 7795 *An*. *gambiae* s.l. and 400 *An*. *funestus* s.l. mosquitoes were assayed in the laboratory by PCR for species ID, which represented all the mosquitoes of this species collected during the entire sampling season. The same mosquitoes were also assessed by ELISA (results shown elsewhere in this paper). All *An*. *gambiae* s.l. mosquitoes were confirmed to be *An*. *arabiensis* (100%). On the other hand, the *An*. *funestus* group consisted of *An*. *funestus* s.s. as the dominant sibling species (84.2%), *An*. *rivulorum* (4.5%), *An*. *leesoni* (1.4%) and a few unamplified samples (9.9%). The unamplified DNA specimen may have been due to improper handling of the specimen prior to PCR, lack of appropriate primers in the PCR assays to detect actual species or incorrect morphological identification before the samples were analysed by PCR.

### Mosquito blood meal sources

A total of 74 mosquitoes with blood meals in their abdomen were also assessed by ELISA to identify the source of their meals. These included 38 *An. arabiensis* and 36 *An. funestus* s.s. The blood-feeding proportions were relatively higher for *An. funestus* [i.e. 9% (36 out of 400) were blood-fed] compared to *An. arabiensis* for which 0.05% (38 out of 7795) were blood fed. Only human blood was identified in all 74 mosquitoes.

### Insecticide susceptibility test results

The insecticide susceptibility test results are summarized in Table 4. Based on the WHO guidelines used here [39], the *An*. *arabiensis* mosquitoes raised from field-collected larvae were found to be susceptible to the carbamate, bendiocarb and to the organophosphate, malathion, but they were resistant to all the pyrethroids tested, i.e. permethrin, deltamethrin, and lambda-cyhalothrin, as well as the organochloride, DDT. Surprisingly, the mosquitoes were also resistant to the other tested organophosphate, i.e. pirimiphos-methyl. This compound has not been used for vector control in this region but is a common agricultural pesticide, and has been used for IRS in northern Tanzania over past 4 years. These insecticide resistance patterns were observed in both Katindiuka and Viwanja Sitini wards, where the tested mosquitoes were obtained. Full susceptibility (100% mortality) was observed in the susceptible Ifakara strain of *An. gambiae* s.s., used here as control. Knock down resistance (*kdr*) assays were not assessed, but a previous study done in villages in the neighboring Ulanga district indicated absence of *kdr* mutation genes [50].

**Table 4:** Susceptibility of wild *An. arabiensis* mosquitoes collected in two wards in the study area, i.e. Katindiuka and Viwanja Sitini, (Fig. 1)

Insecticide tested	Katindiuka ward			Viwanja Sitini ward		
	N	Mortality (%)	Class	N	Mortality (%)	Class
0.75% permethrin	100	37	Resistant	100	37	Resistant
0.05% deltamethrin	100	39	Resistant	100	36	Resistant
0.05% lambda-cyhalothrin	100	32	Resistant	100	34	Resistant
4% DDT	100	41	Resistant	100	65	Resistant
0.1% bendiocarb	100	100	Susceptible	100	100	Susceptible
0.25% pirimiphos methyl	100	54	Resistant	100	70	Resistant
5% malathion	100	100	Susceptible	100	99	Susceptible
Control (untreated paper)	100	0	N/A	100	0	N/A

The assays were conducted using WHO 2013 guidelines for testing insecticide resistance in malaria vectors [39]

In tests conducted on the susceptible *An. gambiae* s.s. (Ifakara strain) used as an additional control, full susceptibility (100% mortality) was observed

### Parity rates of *An*. *arabiensis* and *An*. *funestus* mosquitoes

A total of 109 *An. arabiensis* and 4 *An. funestus* were dissected to determine their parity status. All of these mosquitoes were collected from Katindiuka, Mlabani and Lipangalala wards. Of these, 74.6% of the *An. arabiensis* were parous and 25.7% were nulliparous. On the other hand, all four *An. funestus* were found to be parous (Table 5).

**Table 5:** *Plasmodium* infectious status of primary malaria vectors collected indoors and outdoors in Ifakara town and estimates of the entomological inoculation rates, as contributed by the vector species

	*Anopheles arabiensis*			*Anopheles funestus*		
	Indoors	Outdoors	Overall estimates	Indoors	Outdoors	Overall estimates
Total no. *Anopheles* caught (all traps)^a^	3543	4252	7795	252	148	400
Total no. Trap nights	1792	1780	3572	1792	1780	3572
Total no. *Anopheles* analyzed for CSP	3543	4252	7795	252	148	400
Total no. sporozoite-positive *Anopheles*	0	0	0	0.00	1	1
Annual P*f*EIR	0.00	0.00	0.00	0.00	0.205	0.102
% P*f*EIR contribution by species	0%			100%		

Since all *Anopheles* collected in this survey were analysed for *Plasmodium* infection, no differences were expected between overall P*f*EIR estimations obtained by either the standard or the alternative method as described by Drakeley et al. [13]

^a^In this table, only the alternative method is used to calculate P*f*EIR. The only sporozoite positive mosquito was that captured by human landing catches (HLC), so no adjustments on the other traps were done as they would have marginal effect on overall P*f*EIR estimates, which would be zero nonetheless. Instead, we have considered P*f*EIR estimates without any adjustments and assumed similar trap efficacies. If only outdoor *An. funestus* catches were considered, the P*f*EIR was estimated at double the overall, but these are still very low and only detectable marginally by HLC

### *Plasmodium* infectious rates and malaria transmission intensities

All 8613 *Anopheles* mosquitoes were analysed for *Plasmodium* circumsporozoite protein (CSP) using ELISA. These included 7795 *An*. *gambiae* s.l. (all of which were also determined by PCR to be *An. arabiensis*) and 400 *An*. *funestus* (a majority of which were determined by PCR to be *An. funestus* s.s.). In these ELISA assay, all *An. arabiensis* were uninfected and only one *An. funestus* s.s. mosquito was found to be infected with *Plasmodium*. This infected specimen had been collected outdoors between 03:00 and 04:00 h in Katindiuka ward in August 2015. Coincidentally, this was also the ward with highest malaria vector densities (Table 3). Since all *Anopheles* collected in this survey were analysed for *Plasmodium* infection, P*f*EIR estimations by the standard and alternative methods [13] returned similar results (Table 6). Sporozoite-infected mosquitoes were detectable only by human landing catches, but not any of the other traps (Table 6). Overall P*f*EIR was 0.102 ib/p/yr.

**Table 6:** Comparative estimates of annual P*f*EIR using the two different methods [13], for malaria mosquitoes collected: indoors versus outdoors, in wet versus dry seasons, using different trapping methods, and in the different geographical zones of the study area

Attributes	Category	No. mosquitoes caught	No. trap nights (no. traps per night * no. nights)	Biting rate (no. mosquitoes/no. nights)	No. sporozoites positive	Sporozoite positive rate	P*f*EIR by standard method^a^	P*f*EIR by alternative method ^a^
Location	Indoors	3801	1792	2.12	0	0	0	0
	Outdoors	4394	1780	2.47	1	0.00023	0.205	0.205
Season	Wet (December–May)	7604	1786	4.26	0	0	0	0
	Dry (June–November)	591	1786	0.33	1	0.00169	0.204	0.204
Temperature	Cool (June–September)	706	1190	0.59	1	0.00142	0.307	0.307
	Hot (October–May)	7489	2382	3.14	0	0	0	0
Zones	North and Western area (Viwanja Sitini and Ifakara Mjini)	853	1427	0.60	0	0	0	0
	South and central areas (Lipangalala and Mlabani)	2379	1427	1.67	0	0	0	0
	Eastern peri-urban areas (Katindiuka)	4963	714	6.95	1	0.00020	0.511	0.511
Trapping method	HLC	5197	1200	4.33	1	0.00019	0.304	0.304
	CDC light trap	1894	1192	1.59	0	0	0	0
	Suna trap	1104	1180	0.94	0	0	0	0
Overall		8195	3572	2.29	1	0.000122	0.102	0.102

^a^The standard method calculates P*f*EIR as a product of sporozoites rates and human-biting rates; i.e., C*(Number of sporozoite positive mosquitoes/Number of mosquitoes tested) × (Number of mosquitoes collected/Number of trap nights) × 365(46), where, C is the relative efficiency coefficient for the relationship between CDC-light trap catches and human landing catches. The alternative method calculates P*f*EIR the ratio of sporozoite positive mosquitoes and number of nightly catches; i.e., C*(no of sporozoite positive mosquitoes/number of trap nights) × 365(14). Though the sporozoite rates are varied, the P*f*EIR estimates are same, primarily because all *An. gambiae* and *An. funestus* were analysed

### Association between household characteristics and malaria vector densities

Of the 110 houses observed, 94.5% were constructed with brick walls and 5.5% had mud walls. Likewise, 97.3% of the houses had metal roofs and 2.7% had thatched roof. The houses had an average of 5.3 windows, ranging from 2 windows in small houses to 17 windows in the largest houses. The average number of outlet doors was 1.7, ranging from 1 to 7 doors. Half of the windows across the study area were covered with netting screens (51.8%), while the rest had wood or metal on the windows (17.3%), bricks (16.4%) or cardboard and clothes (13.6%). Only one house had completely uncovered windows (0.09%). The type of window cover had an effect on indoor densities of the primary malaria vectors, as lower indoor densities were observed in houses with netting screens on the windows compared to houses with other types of covers (Table 7).

**Table 7:** Indoor and outdoor densities of the malaria vector species, *An. arabiensis* and *An. funestus* mosquitoes in houses with different characteristics in Ifakara town and its adjacent wards

House characteristics	No. houses (N)	Mean no. *An. arabiensis* per house [LCI–UCI]	Mean no. *An. funestus* per house [LCI–UCI]	t-test P-value
Indoor mosquito densities (association of vector densities with electricity)				
Without electricity	53	59.1 [27.9–90.3]	4.63 [2.2–7.1]	<0.05
With electricity	57	7.3 [4.6–10.0]	0.18 [− 0.03–0.4]	
Outdoor mosquito densities (association of vector densities with electricity)				
Without electricity	53	67.2 [18.0–116.4]	2.6 [0.2–4.9]	<0.05
With electricity	57	12.0 [1.7–22.6]	0.3 [0.1–0.5]	
Indoor mosquito densities (association of vector densities with window covering)				
Windows covered with netting screen	57	14.8 [7.0–22.6]	1.6 [0.4–2.8]	<0.05
Windows covered with wood or metal	19	24.0 [18.1–29.9]	0.8 [− 0.2–1.8]	
Windows covered with bricks	18	36.1 [11.5–83.7]	3.6 [− 1.3–8.5]	
Windows covered with cardboard/clothes	15	75.3 [4.3–146.3]	5.4 [0.2–10.7]	
Windows uncovered	1	471.0 [471.0–471.0]	3.0 [3.0–3.0]	

All (100%) of households had at least one insecticide-treated net (ITN). It was also observed that 51.8% of the houses had electricity while 48.2% did not. Electricity was mostly in the Ifakara Mjini, Lipangalala and Viwanja Sitini wards. All the sampled houses in Katindiuka ward had no electricity, and only 7 of the 22 households in Mlabani had electricity. Most of the houses in the peri-urban ward of Katindiuka were also surrounded by small rice fields and water ponds, especially during the wet season while houses in the urban settings of Ifakara Mjini and Viwanja Sitini wards were generally surrounded by small businesses such as small shops, vegetable stalls and *bodabodas* (motorcycle taxis) (Fig. 5). Lower mosquito densities were observed in houses with electricity compared to houses without electricity (Table 7). A great deal of brick-making activities was also observed in the dry season which resulted in a lot of pits that may have provided adequate breeding habitats for mosquitoes. Lastly, only one household had cattle, but 49 of the households (44.5%) had chickens or ducks.

## Discussion

Africa is the world’s most rapidly urbanizing continent [1, 2]. Currently about 40% of the African population resides in urban settings, but that proportion is expected to reach 56% by 2050 [1, 2]. Malaria, like most other infectious diseases, is negatively impacted by urbanization [7–10], since urban settings are characterized by better access to health care services, better housing and reduced availability of larval habitats [8–10]. In Tanzania, the urban population is increasing at 5.4% per annum. Tanzanian urban population was estimated at 5.7% in 1967 [15], but it had increased to 22.6% in 2002 [4], 29.1% in 2012 [51] and is estimated to be 33% in 2018 [3].

This current study focused on one of the fast-growing towns in Tanzania (i.e. Ifakara), and where demographic, socio-economic and epidemiological transitions are strongly evident. Ifakara town, in the Kilombero river valley is surrounded by rain forests, wetlands, savannah lands making it an attractive setting for both farmers and pastoralists. More importantly, as a town in the middle of an expansive low-lying flood-plain, only eight degrees south of the Equator, the area presents a unique epidemiological profile with very high malaria transmission in surrounding areas.

Though there have been several entomological surveys in the villages across the Kilombero valley over the past three decades [12, 52–54], there have been limited investigations within the Ifakara town centre itself, or in its closely adjacent wards. The most recent examination of malaria transmission in the town area was completed more than 15 years ago by Drakeley and colleagues [13]. Over the years, since the study by Drakeley et al. in early 2000s, the area has experienced significant increases in population concurrent with urbanization, due to in-migration by small business owners, farmers and pastoralists (Mayor of the Ifakara Town Council—Personal Communication). This current study presents the first reassessment of transmission in this community in nearly two decades.

A year-long entomological surveillance of malaria vectors was conducted in the five administrative wards making up the Ifakara town, an area with a total population estimated at 70,000 people. The magnitude and biting patterns of main malaria vector species, the levels of ongoing transmission, as well as the key factors driving the transmission in the town were identified. A general observation was that densities of malaria vectors within the area was found to be much lower compared the vector densities usually observed in nearby rural villages on both sides of the Kilombero river [21, 22]. Overall P*f*EIR was estimated to be 0.102 ib/p/yr, which was entirely driven by *An. funestus* and only detectable by HLC. This was more than 99% decrease from the P*f*EIR reported by Drakeley et al. in 2003 from the same area. The P*f*EIR estimates are also much lower than those recently observed by Kaindoa et al. in the neighbouring rural villages of Ulanga district (P*f*EIR = 18.45), about 20 km south of Ifakara town [21]. Some of the factors that could be attributed to this dramatic decline could be urbanization [7, 8, 10], the universal bed net coverage campaigns that started in 2004 [17, 55], improved housing [56] as well as improved diagnosis and treatment [16]. More than 90% of the houses in this study area now have brick walls and metal roofs, more than 50% had screened windows, and all (100%) had at least one bed net (Finda et al., unpublished). Unfortunately, this study was limited by the lack of aerial maps for the study area from 15 years ago, which would have enabled more detailed comparison of housing developments in the area. Nonetheless, these improvements, coupled with the ongoing large-scale use of long-lasting insecticide-treated nets (LLINs) and other malaria control interventions, as well as the changes in potential mosquito breeding habitats, it can be expected that malaria transmission should plummet even further.

The ease of detecting *Plasmodium* circumsporozoite proteins in mosquitoes using simple ELISA assays and the simplicity of calculating entomological inoculation rates (P*f*EIR) has made this approach fairly popular for assessing malaria endemicity and transmission intensity [44, 57]. It requires only simple measures of mosquito biting rates and sporozoites positivity rates, and is therefore a more direct method of measuring transmission intensity compared to other measures such as parasite incidence or prevalence and has been used widely for assessing impact of vector control programs in Africa [57, 58]. This method estimates the number of bites by infectious mosquitoes per person per unit time. It is the product of the “human biting rate”—the number of bites per person per day by vector mosquitoes—and the fraction of vector mosquitoes that are infectious [44]. However, despite its widespread use, there have also been concerns that P*f*EIR estimates may be inconsistent, and that there can be large variabilities in P*f*EIR estimates from the same area [57, 58]. Such variations are heightened by the differences in methods of measurement [57, 58] and the lack of clear relationship between P*f*EIR estimates and malaria parasite prevalence of incidence rates in localities with low transmission intensities [59, 60]. Besides, at very low P*f*EIR ranges, available methods are imprecise and there are very slim chances of getting mosquitoes at a precise time when innoculation takes place [60]. Therefore, the very low P*f*EIR estimates observed here do not necessarily mean absense of local transmission, but rather the lack of effective measurement methods.

In this study, only the human landing catches method was able to detect sporozoites positive mosquitoes. On the contrary, neither the CDC light traps nor the Suna^®^ traps caught any infected mosquito. This suggests the need for much more sensitive tools and approaches for measuring human exposure to malaria parasites in conditions such as this, where parasite densities in mosquitoes have become too low. Nevertheless, it has also been argued that if similar methods are used to consistently estimate transmission over time, such as this study has done, and if adequate stratification is performed, then interventions that drive malaria transmission to P*f*EIR < 1, could be effective in achieving local elimination [61]. In this survey, the overall P*f*EIR of 0.102 ib/p/yr was indeed far below the threshold of 1 ib/p/yr, beyond which sustained local efforts could lead to complete disruption of local malaria transmission.

Going forward, improved measurements are therefore a critical component of the malaria elimination agenda [62]. It will thus be essential to deploy improved measurement methods that can assess both the burden and the transmission of malaria pathogens in such situations so as to support further efforts for elimination. It would also be important to couple such an entomological survey with a malaria parasite prevalence study, using methods capable of detecting low-level parasitaemia so as to more comprehensively understand the residual malaria epidemiology in the area.

Within the Ifakara town, the highest density of malaria vectors was observed in Katindiuka and Mlabani (Fig. 6) wards, which were also the most rural of the five wards. Households in Katindiuka and Mlabani wards were surrounded largely by rice paddies and water ponds (Fig. 5), and nearly all the houses in these wards did not have electricity. Brick-making activities were fairly common in these areas in the dry season which resulted in a lot of pits with standing water in the rainy season, which may have provided adequate breeding habitats for mosquitoes. Although nearly all of the households surveyed were made with bricks and metal roof, majority of the households in Mlabani and Katindiuka lacked electricity, hence they were in the dark through most of the night, which may also have provided a suitable environment for host seeking mosquitoes [63].

In the study done by Drakeley et al, 91.5% of all *An. gambiae* s.l. were *An. arabiensis* while only 8.5% were *An. gambiae* s.s., none of which were sporozoites-positive. However, several studies done since the Drakeley et al. study have documented absence of *An. gambiae* s.s. from the Kilombero river valley, mainly attributable to the use of LLINs [22, 52, 54, 64]. While the most abundant malaria vector species was found to be *An. arabiensis*, it was *An. funestus* that was found to drive all of the transmission in the town. Only 400 *An. funestus* mosquitoes were collected compared to 7795 *An. arabiensis*, yet the only *Plasmodium* infected mosquito was an *An. funestus*. Though only a single infected *An. funestus* might be considered too few to conclude on the dominance of the species, evidence from neighboring villages suggest this is most likely the case. Similar patterns of dominance were indeed observed by Kaindoa et al. [21], who showed *An. funestus* to carry more than 80% of all the malaria transmission in neighboring villages in the Kilombero Valley. All blood-fed mosquitoes were found to contain human blood, and this can be explained by the absence of big livestock such as cows and goats in the urban settings, hence humans were the most available source of blood meals for the vectors. This could suggest that household-based interventions could still be effective in targeting malaria vectors in the Ifakara town. However, the actual blood-feeding proportions were relatively higher for *An. funestus* (i.e. 9% (36 out of 400) were blood-fed) compared to *An. arabiensis* for which 0.05% (38 out of 7795) were blood fed. This further demonstrates the importance of *An. funestus*.

Relatively high parity rates were observed; over three quarters of all the mosquitoes dissected were found to be parous, a fact that also emphasizes the need for interventions that prevent man–vector contact to limit local transmission. On the basis of parity rates and human blood index, both *An. funestus* and *An. arabiensis* can be considered important vectors in the area. However, based on actual contribution to P*f*EIR estimates, *An. funestus* is likely mediating most of the on-going residual transmission in both the wider valley and in Ifakara itself. In this study, *An. funestus* densities were also observed to be higher in the dry season, which is the time of the year that people are most relaxed with regards to protection against mosquitoes.

There was evidence of phenotypic resistance in *An. arabiensis* to permethrin, deltamethrin, lambda cyhalothrin, pirimiphos methyl and DDT. However, the same mosquito populations from the study villages were found 100% susceptible to bendiocarb and malathion. The most surprising aspect of this specific finding was that the organophosphate, pirimiphos methyl has not previously been used in the area for vector control. It is however widely used in agriculture (Matowo et al., pers.comm.), which could be the source of this resistance pressure exhibited here. Nonetheless, the resistance profile of the mosquitoes collected here is worrying as it signals that there is a very narrow set of insecticide options now available for malaria vector control. One minor limitation with this aspect of the study was that the resistance assays were conducted only in two of the five wards, primarily due to availability of *Anopheles* larvae at the time of the tests. These findings can however still be considered fairly representative of the whole study area, and concur with other studies that have been done in surrounding villages in the valley [21, 50].

Finally, the major decreases of malaria transmission seen are indeed impressive but should not be taken as a sign of impending local elimination. Instead, it should be interpreted as evidence that local elimination is possible given multisectoral approaches that combine house improvement to other technologies such as LLINs and effective case management, with proper diagnostics and medicines. The authors propose a parasitological survey to enable assessment of actual malaria cases prevalence and incidence rates, and also to examine in greater detail the actual rates of importation. Households should be encouraged to continue use of their long-lasting insecticide treated nets and to visit health facilities urgently whenever they experience any fevers. Given the significance of house improvement, these efforts too, should be encouraged to further reduce exposure to malaria and other mosquito-borne infections.

## Conclusion

Malaria transmission intensity in Ifakara town and its surrounding environment has declined by over 99% over the past 15 years, reaching levels nearly undetectable with current entomological methods. Over a total of 3572 trap-nights, only one *Plasmodium*-infected *Anopheles* was found, an *An. funestus* mosquito caught outdoors in the peri-urban ward of Katindiuka. The overall P*f*EIR of 0.102 ib/p/yr is far below the threshold of 1 ib/p/yr, beyond which sustained local efforts could lead to complete disruption of local malaria transmission. This decline is likely associated with urbanization, improved housing, insecticide-treated nets and improved case management. House designs have vastly improved in the area and more than 90% of houses currently have brick walls and/or metal roofs, and more than half have screened windows. The remaining risk of *Anopheles* biting and malaria transmission in the valley is now mostly localized in the more rural ward adjacent to the town centre indicating that urbanization does indeed play a role in the control and possible elimination of malaria. On the basis of parity rates and human blood index, both *An. funestus* and *An. arabiensis* can be considered important vectors in the area. However, based on actual contribution to P*f*EIR estimates, *An. funestus* is likely mediating most of the on-going residual transmission in both the wider valley and in Ifakara itself, despite occurring in comparatively lower densities than *An. arabiensis*. Insecticide resistance has increased significantly, particularly against pyrethroids, organophosphate, pirimiphos-methyl and the organochloride, DDT, a situation which will clearly limit insecticidal options for malaria prevention. It is therefore possible that new cases reported in this area are now likely arising from infections outside the area than from within. It is also likely that concerted efforts could cause further decreases, possibly achieving complete disruption of local transmission in this area. Future surveys of malaria should deploy improved approaches for measuring transmission and parasite infection reservoirs in areas with such low transmission intensities. The authors specifically propose a parasitological survey to assess actual malaria prevalence and incidence rates, and estimate actual proportions of local and imported cases in the area.
